# Towards making efficient use of household resources for appropriate prevention of malaria: investigating households’ ownership, use and expenditures on ITNs and other preventive tools in Southeast Nigeria

**DOI:** 10.1186/1471-2458-14-315

**Published:** 2014-04-05

**Authors:** Obinna Onwujekwe, Enyi Etiaba, Nkoli Uguru, Benjamin Uzochukwu, Alex Adjagba

**Affiliations:** 1 Department of Pharmacology and Therapeutics, Health Policy Research Group, College of Medicine, University of Nigeria Enugu-Campus, Enugu, Nigeria; 2 Department of Health, Administration and Management, College of Medicine, University of Nigeria Enugu-Campus, Enugu, Nigeria; 3 Department of Preventive Dentistry, College of Medicine, University of Nigeria Enugu-Campus, Enugu, Nigeria; 4 Department of Community Medicine, College of Medicine, University of Nigeria Enugu-Campus, Enugu, Nigeria; 5 SIVAC Initiative, Agence de Medecine preventive (AMP), Paris, France

**Keywords:** Malaria, Socioeconomic status, ITNs, Other preventive tools, Equity

## Abstract

**Background:**

Many households own, use and spend money on many malaria preventive tools, some of which are inappropriate and ineffective in preventing malaria. This is despite the promotion of use of effective preventive methods such as Insecticide treated nets (ITNs) and indoor residual house spraying (IRHS). The use of these ineffective methods imposes some economic burden on households with no resultant reduction in the risk of developing malaria. Hence, global and national targets in use of various effective malaria preventive toools are yet to be achieved in Nigeria. This paper presents new evidence on the differential use and expenditures on effective and non-effective malaria preventive methods in Nigeria.

**Methods:**

Semi-structured interviewer administered pre-tested questionnaire were used to collect data from 500 households from two communities in Enugu state, Nigeria. The two study communities were selected randomly while the households were selected systematically. Information was collected on demography, malaria status of children under 5 within the past month, types of malaria preventive tools used by households and how much was spent on these, the per capita household food expenditure and assets ownership of respondents to determine their socio-economic status.

**Results:**

There was high level of ownership of ITNs (73%) and utilization (71.2%), with 40% utilization by children under 5. There were also appreciable high levels of use of other malaria preventive tools such as window and door nets, indoor spray, aerosol spray and cleaning the environment. No significant inequity was found in ownership and utilization of ITNs and in use of other preventive methods across socioeconomic groups. However, households spent a lot of money on other preventive tools and average expenditures were between N0.83-N172 ($0.005-$1.2) The richest households spent the most on window and door nets (P = 0.04).

**Conclusion:**

High levels of use and expenditure on ITNs and other malaria preventive tools exist. A programmatic challenge will involve designing ways and means of converting some of the inefficient and inappropriate expenditures on many ineffective malaria preventive tools to proven cost-effective methods such as ITNs and IRHS. This will help to achieve universal coverage with malaria preventive tools.

## Background

Malaria has a significant impact on the health of infants, young children, and pregnant women worldwide. More than 800,000 African children under the age of five die of malaria each year. Malaria also contributes to malnutrition in children, which indirectly causes the death of half of all children under the age of five throughout the world. Fifty million pregnant women throughout the world are exposed to malaria each year. In malaria-endemic regions, one-fourth of all cases of severe maternal anaemia and 20 percent of all low-birth weight babies are linked to malaria [1].

In Nigeria, malaria is responsible for 60% of outpatient visits, the leading cause of under-five mortality contributing 33% of all childhood deaths and 25% infant mortality [2]. A child will typically suffer from malaria about 3–4 times in a year causing absence from school in children of school age. This in turn impedes their educational and social development and subsequently impacts negatively on the country’s future human resources [2]. This also poses significant economic burden on the country; about 132 billion naira (USD $870 million) is lost every year in form of malaria prevention, treatment costs and loss of overall economic productivity [3].

Malaria is a difficult disease to control largely due to the highly adaptable nature of the vector and parasites involved. While effective tools have been and will continue to be developed to combat malaria, inevitably, over time the parasites and mosquitoes will evolve means to circumvent those tools if used in isolation or used ineffectively [1].

Vector management tools such as insecticides, environmental modification, and bed nets have contributed greatly to successful malaria control efforts historically, but have faced setbacks in recent years due to factors such as the emergence of insecticide resistance in mosquitoes [1]. Methods other than Indoor Residual House Spraying (IRHS) and Insecticide-Treated Nets such as long-lasting insecticide-treated nets (LLINs); are less universally applicable, and so depend more on local technical expertise. These other methods include: larviciding, or chemically treating known mosquito breeding sites, environmental management, or draining swamps and other sites where mosquitoes might breed, personal protection measures, such as bug repellents, and window screens, Fogging, or covering large areas of land with insecticide (this method can lead rapidly to mosquito resistance and adverse environmental effects). However, it has been reported that these methods can be quite useful in conjunction with the two primary preventive interventions, and sometimes replace them in areas of low transmission [4].

The control of malaria in Nigeria adopts a holistic approach through case management and prevention. One of the objectives of the National Malaria Strategic Plan 2009–2013 amongst others, is to gradually scale up spraying using IRHS to cover 20% of households nationwide (or almost seven million households) by 2013 [5]. The target for distribution of ITNs is to meet the MDG target of 80% coverage by 2015 [5]. In order to achieve these targets, the government has been propelled to undertake many interventions including the free distribution of ITNs (the long-lasting insecticide treated nets-LLINs) to pregnant women and children aged under-five years. However, despite all these efforts, global and national targets to achieve usage of various effective malaria preventive tools are yet to be met in Nigeria.

Many households own, use and spend money on many non-drug malaria preventive tools, some of which are inappropriate and ineffective in preventing malaria. This is despite the promotion of use of ITNs. Besides ITNs, other non-drug malaria preventive tools that are utilized include Indoor residual House Spraying (IRHS), larviciding and environmental management [5].

Geographic and socio-economic inequities have been reported in the control of malaria, with rural dwellers and poor households less likely to prevent and appropriately treat malaria [6]. The higher cost of malaria episodes on the poor especially those in the rural areas is exacerbated because they are less likely to purchase malaria preventive tools, such as insecticide treated nets or seek prompt effective treatment with an episode of malaria in contrast to their urban dwelling or richer counterparts [7,8]. Instead they use cheaper traditional malaria prevention methods like burning or drinking of local herbs, mosquito coils, cleaning of environment which offer only partial protection [9].

This paper presents new information about the levels of ownership, use and expenditures on ITNs and other malaria preventive tools amongst different households. It shows the levels of use and expenditures on both effective and non-effective malaria non-drug preventive methods in Nigeria.

## Methods

### Study area

The study sites are Achi and Oji urban communities located in Oji-River local government area of Enugu state, Southeast Nigeria, with an average malaria incidence rate of 15%. Achi community is located 5 kilometers from the local headquarters called Oji-River and 45 kilometers from the state capital, Enugu. It has an estimated population of 46,112 people and is divided into 12 villages. Oji urban, with a population of 14,026, is made up of 4 political wards. Both communities are linked to Oji-River local government headquarters by a single lane road covered with asphalt, which presents a formidable challenge for users especially during the raining season. Dirt roads and bush paths provide means of access to the interiors in the villages [10]. Both LGAs are populated by one dominant ethnic group though other ethnic groups from various parts of the country reside there as well. The predominant occupation and religion are subsistence farming and Christianity respectively while the Igbo language is the most widely spoken local language [11].

### Study design, sampling strategy, sample size and data collection

This was a descriptive cross-sectional study involving a survey of households with children under 5 years old. The inclusion criteria were households with children under 5 years in selected communities. Pre-tested interviewer administered semi-structured questionnaires were used to interview the primary care giver in the households.

The two communities used in the study were randomly selected from a list of all the six communities in Oji LGA using the balloting technique. A list of all the households with children under five years in the two communities were compiled using the Primary Health Care (PHC) house numbering system. This was done in order to get a sample frame of the households with children under five years. An adequate sample size was computed based on a power of 80% and confidence interval of 95%. A systematic random sampling technique was used to select 500 households for the study. Every k^th^ household (in this case 2nd) from the list of enumerated households was selected until the sample size of 500 was completed. The primary caregiver or representative in each of these households was interviewed after obtaining informed consent.

Data was collected from the household respondents (primary caregivers or representatives) by field workers using a pre-tested interviewer administered questionnaire. Having obtained written informed consent, information was collected on personal data and demographics of respondent, malaria status of children under five years within the past one month, ownership and utilization of ITNs and untreated bed nets (those who slept under a net the night before the interview), use of other malaria preventive tools as well as the costs expended by the respondents on purchasing these bed nets and other malaria prevention methods. Finally information was collected on the per capita household food expenditure and assets ownership of respondents to determine their socio-economic status.

### Other Vector control tools included in the study besides ITNs and IRHS

These include: Aerosol sprays which are commercially sold vector repellents sold in small cans; untreated window and door netting, which are windows and doors covered with a wire mesh to prevent access of vectors into the household; mosquito coils which are solid chemical substance burnt like incense which releases fumes believed to be repellent to mosquitoes; and other mosquito repellents, which are various forms of chemical substances also believed to repel mosquitoes.

### Data analysis

Tabulations, descriptive statistics and nonparametric tests were the major data analytic procedures that were used. A pooled data from the two communities were used to examine the type of malaria prevention methods that households sought, ownership and utilization of ITNs, differences in cost incurred by different households and inequity in total costs. In equity analysis, an asset based and household expenditure was used to categorize the households into SES quintiles: rich, least poor, poor, very poor and most poor. Principal components analysis (PCA) was used to generate the index [12] that was used to investigate the equity implications of the findings. Information on ownership of a radio, bicycle, motor car, grinding machine and motorcycle together with the weekly per capita cost of food was used to generate the SES index. Pearson’s Chi squared test was used to test significance of associations and P value of <0.05 accepted as significant. Equity ratios between the poorest and richest quintiles (Q1:Q5) were also computed.

### Note

158 Naira=1 US$

### Ethical considerations

Ethical clearance was obtained from the ethical review board of the University of Nigeria Teaching Hospital Enugu.

## Results

### Socio - demographic characteristics of respondents

Of the 500 respondents surveyed in the community, 96.8% were females and the mean age of respondents was 33 years (Table 1). A little over half (57.6%) of the households had a child/children with malaria at the time of or one month prior to the survey. The occupation of the respondents varied widely from petty trading/Artisan to employment in a private organization.

**Table 1:** Socio-demographic characteristics of respondents

**Characteristics**	**Measurement**
**Sex: n (%)**	
Female	484 (96.8)
Male	16 (3.2)
**Age in years: mean (sd)**	33 (9.2)
**No. of people in a household: mean (sd**)	5 (1.85)
**Occupation: n (%)**	137 (27.4)
Petty trading/Artisan	109 (21.8)
Subsistence farmer	48 (9.6)
Large scale entrepreneur	39 (7.8)
Self employed	37 (7.4)
Government worker	12 (2.4)
Employed in a private organization	39 (7.8)
Unemployed	79 (15.8)
Others	

Note: sd = standard deviation.

### Ownership, Utilization and Maintenance of ITNs

Most of the survey households owned at least one bed net, of which 71.8% were ITNs (Table 2). It was found that only a minimal number; 9(1.8%) of households purchased ITNs while the rest acquired their nets through the free bed net distribution programme (Table 2). Almost all the households that owned nets used them (71.2%). Table 2 shows that 40% of use was by children under five years, whilst 28.4% was by adults. No household had treated, re-treated or mended their nets in the last 6 months.

**Table 2:** Ownership, Utilization of Bed Nets

**Variable**	**n**	**%**
**Ownership of mosquito net**	**365**	**73**
Free ITN	350	70
Free untreated net	6	1.2
Bought ITN	9	1.8
**Utilization (who slept under net)**	**356**	**71.2**
Children	200	40
Adults	142	28.4
Youth	7	1.4
Others	7	1.4

### Use and costs of other non-drug malaria preventive methods

Data on utilization of other preventive methods was only available from 271 (54.2%) households but a majority of these households utilized more than one method (Table 3). Window and door nets as a method of prevention was the most expensive and used by about a fifth of the households, while smoke which cost nothing was the least utilized. Cleaning the environment and clearing of vegetation around households were also popular methods of malaria prevention, used by 42.4% and 28.8% respectively. The mean cost of other preventive methods was valued at $1.7 USD (259 Naira) per month.

**Table 3:** Utilization and Cost of Other Preventive Methods

**Method**	**Utilization(n)**	**Cost [Naira(std)]**	**Cost-USD**
Cleaning environment	212 (42.4)	20.2 (82.4)	0.13
Clearing of vegetation	144 (28.8)	14.5 (120.6)	0.1
Window & door net	92 (18.4)	172.6 (54.2)	1.2
Indoor spray (IRHS)	46 (9.2)	138.5 (181.3)	0.92
Aerosol spray	36 (7.20)	136.9 (193.1)	0.91
Burning mosquito coil	35 (7.0)	35.2 (52.1)	0.2
Other Mosquito repellants	28 95.6)	109.6 (165.6)	0.73
Growing plants	7 (1.4)	0.83 (6.45)	0.005
Ingestion of herbs	4 (0.8)	15.7 (119.2)	0.1
Smoke	2 (0.4)	0 (0)	0

### Net ownership and utilization by socio-economic status (SES) quintile

Table 4 shows that ownership of ITNs was almost evenly distributed among the five SES quintiles; the poorest quintile owned the least nets (18.4%) while the richest quintile owned the most nets. The middle quintile (Q3, poor) owned more nets than the least poor quintile (Q4) but these differences were not statistically significant (P > 0.05).

**Table 4:** Ownership and Utilization of ITNs by SES

**SES**	**Net Ownership n (%)**	**Utilization n (%)**
Q1: most poor	67 (18.6)	65 (18.4)
Q2 :very poor	71 (19.7)	68 (19.3)
Q3 :poor	73 (20.3)	72 (20.4)
Q4 :least poor	69 (19.2)	69 (19.5)
Q5 :rich	79 (22.1)	79 (22.4)
**Q1:Q5**	**0.85**	**0.82**
**X**^**2 **^**(P value)**	**3.66 (0.45)**	**4.88 (0.76)**

Utilization of ITNs by households followed a similar pattern as ownership, with the highest utilization by the richest households (Q5) and least use by the poorest group (P > 0.05).

### Socio-economic status (SES) differences in use of other malaria prevention methods

The poorest SES group (Q1) is the only group that uses smoke as a malaria prevention tool and also uses clearing of vegetation more than other SES groups. The richer SES groups significantly use more window and door netting as a method of prevention (Table 5), but there is no significant difference in the use of the other methods of malaria prevention between the SES groups.

**Table 5:** Socio-economic status (SES) differences by prevention method

**Variable**	**Q1**	**Q2**	**Q3**	**Q4**	**Q5**	**X**^**2 **^**(p value)**
Other Mosquito repellants	6 (21.4)	5 (17.9)	4 (14.3)	9 (32.1)	4 (14.3)	2.51 (0.64)
Smoke	2 (100)	0	0	0	0	7.5 (0.11)
Clear vegetation	39 (28.3)	27 (19.6)	28 (20.3)	24 (17.4)	20 (14.5)	7.4 (0.12)
Eat/drink herbs	1 (25)	2 (50)	1 (25)	0 (0)	0 (0)	1.3 (0.9)
Burn mosquito coil	6 (17.1)	7 (20)	4 (11.4)	14 (40)	4 (11.4)	7.3 (0.12)
Cleaned environment	33 (16.3)	39 (19.2)	34 (16.7)	41 (20.2)	56 (27.6)	4.9 (0.29)
Aerosol spray	6 (16.7)	10 (27.8)	4 (11.1)	5 (13.9)	11 (30.6)	4.0 (0.4)
Indoor spray (IRHS)	8 (17.4)	9 (19.6)	8 (17.4)	12 (26.1)	9 (19.6)	0.7 (0.9)
Grow plants	1 (14.3)	1 (14.3)	3 (42.9)	1 (14.3)	1 (14.3)	2.8 (0.58)
Window and door net	9 (10.1)	14 (15.7)	16 (18)	22 (24.7)	28 (31.5)	8.4 (0.04)

### Utilization and cost of other preventive methods by wealth quintile

Over 50% of households in each of quintiles Q1-Q4 utilized one or more of other preventive methods, while the richest quintile was slightly less (Table 6). The significant difference in use of window and door net (Table 5) disappeared when lumped with other methods (Table 6), though equity ratio is minimally pro-poor.

**Table 6:** Utilization and Cost of other Preventive Methods by Wealth quintile

**SES**	**No. that used (n)**	**Mean cost (std)**	**Mean cost (std)**
		**Naira**	**USD**
Q1	51	148.63 (233.13)	0.96 (1.51)
Q2	59	160.34 (258.10)	1.04 (1.67)
Q3	50	166.20 (295.0)	1.07 (1.91)
Q4	64	285.94 (441.17)	1.85 (2.86)
Q5	47	569.13 (797.85)	3.69 (5.17)
**Q1:Q5 ratio**	**1.08**		
**X**^**2 **^**(P value)**	**6.77(0.14)**		**0.4 (0.9)**

## Discussion

This study shows that almost three of every four households own at least one ITN. This represents a high level of ownership given the national targets and is likely due to the free net distribution by the state government to mostly pregnant women and children under-five years, although many people still purchase ITNs from pharmacy shops and from other markets to complement the free nets distributed to pregnant women and children.

It is possible to achieve national targets despite the fact that the ‘law of inverse equity’ [13] is applicable here and the law describes a situation where the rich capture more of the benefits of publicly provided services when coverage is low, and as coverage increases the poor will then start benefiting equally. This also implies that the national target of net ownership is achievable if campaigns and ITN distributions are sustained. In 2005, a study that was conducted in this same study area found that none of the respondents had ever bought or owned an ITN and only 15.3% owned ordinary nets [11]. Net ownership may have been biased upwards by a net distribution exercise that was carried out in the area shortly before the study - during the Maternal and Child health (MCH) week. The current level of coverage, despite the bias, shows progress given that at the beginning of the RBM program in Nigeria, ITNs were barely existent [14].

There is also a high level of ITN utilization, almost the same as ownership, and children under 5 years constitute a significant proportion. The current level of utilization shows progress given the national baseline of 0% in 2000 and the 2003 NDHS figure of 1.2% [15]. It is possible and not uncommon for more than one person in a household to sleep under a net, so there is the additional benefit that may not have been captured in this study. This study was carried out during the rainy season when the weather is cool and more comfortable to sleep under the nets, as also shown in a study in Ghana, which showed a 99% use of nets in the peak of rainy season [16]. This can be contrasted with a low utilization rate during the hot dry season when people are more prone to sleeping outside the nets [17].

Net purchasing is not very popular in this area as seen from earlier studies [18,19]. It may then follow that a rise in net ownership over time may be most likely due to the free net distribution by some health facilities in this area. Studies in Nigeria, Senegal, Uganda and Zambia showed that reduced prices of ITNs amongst other factors resulted in impressive gains in awareness, ownership and use of ITNs [20]. In Afghanistan, knowledge and value of ITNs was high, but the price of ITNs and distribution strategies were barriers to ownership and utilization [21].

The study shows equity in utilization (as well as ownership) of ITNs among the socio-economic quintiles and this is crucial as actual protection from malaria transmission depends on usage rather than ownership of ITNs. Free net distribution appears to have bridged the inequity in net ownership across the socio-economic groups. In the early years following the Abuja malaria summit, there was wide ranging inequity in net distribution and ownership across many African countries [22]. A Tanzanian study noted almost perfect equality in net ownership and usage following a free RBM net distribution programme [23], as was also found in this study. Another study showed marked inequity across the socio-economic quintiles, the abolition of which required mass or targeted free net distribution, though there was a risk of damaging the existent effective commercial market [24].

A little over half the study population used one or more of other preventive tools besides ITN, and of these, window and door nets were the most popular followed by IRHS. Besides eating and drinking of herbs most other tools used were one form of environment management or the other. These tools are not nationally coordinated as there are no guiding policies in place yet.

In general, the poorer socio-economic groups use less expensive malaria prevention methods than the richer SES groups. This could be because of the cost implications; though they may actually be aware of the benefits of malaria prevention but the financial implications may be so daunting that the tendency to use a prevention method that costs virtually nothing is more attractive to them. Most of the people in this group are also subsistence farmers who probably live in homes without proper doors or windows and so using methods like aerosol spray or mosquito coil will be ineffective and in the long run turn out to be a waste of the little resources that they have [9]. These low levels of expenditures indirectly mean that the poor will be more exposed to mosquito bites and hence are more likely to suffer from malaria. This finding is similar to that observed in another study showing that malaria may adversely affect economic activity and lead to poverty, but that it is also possible that the poor are less able to protect themselves from malaria and less able to seek effective prevention and treatment and therefore experience greater morbidity from the disease [9].

It was intuitively plausible that the richest quintile in this study spent the most on other preventive tools; the significant difference in use of window and door nets by the richest quintile disappeared when this was lumped together with other preventive methods giving no statistically significant association between socio-economic status and cost as well as usage of other preventive tools. This is surprising because the other malaria prevention methods are considerably cheaper than buying bed nets but still the poorer SES group do not spend on these methods. Incidentally, the study site has a good complement of traditional practitioners who perpetuate different traditional or herbal practices which are most likely cheap and will compete with the orthodox method of preventing malaria. The richer SES groups spend more on window and door netting when compared to the lower SES. This finding is similar to a previous qualitative research conducted in the study area which also showed that window nets were preferred to bed-nets, but at the time the cost was the deciding factor in type of nets owned by households [19]. In Sudan, where the government used to fund IRHS and not ITNs, it was found that IRHS was more utilized [25]. Across some other countries, poorer households have been shown to be less likely to pay for health services, and when forced to by ill- health, their minimal resources are further depleted [26,27].

A limitation of the study was that it did not compare urban and rural dwellers because that would give further insight on inequities that may exist. The study did not also investigate how many bed nets were owned and used by each household, which might actually bring to light more expenditures being made on prevention. In addition, only households with under-five children were included in this study even though other family members also slept under nets. This may explain the high proportion of net ownership found in this study since this vulnerable group is more likely to own nets, being a priority group in the RBM initiative. This may have caused an overestimation in this population. However, this is a vulnerable group, so it should be expected that coverage in households with no child (children) under 5 years may well be lower than obtained in this study. It would also be informative in the future to look at pattern of usage of ITNs and other preventive methods in pregnant women who are another vulnerable group.

## Conclusions

In conclusion, this study shows that there were high levels of use and expenditure on ITNs and other malaria preventive tools. It was also found that although IRHS was supposedly implemented free for some households, some households paid for the service. Altogether, the high levels of use of ITNs and other malaria preventive tools may not proportionately translate to reduction in incidence of malaria since some of these tools have not been proven to be effective and may impact negatively in reducing the economic burden of malaria. The free distribution of ITNs by the government may have succeeded in ensuring appreciable levels of equity in net ownership and use. However, a programmatic challenge will involve designing ways and means of ensuring sustainability in high coverage levels with ITNs and IRHS and converting some of the inefficient and inappropriate expenditures on many ineffective malaria preventive tools to the proven cost-effective methods. This will help to achieve universal coverage with malaria preventive tools.

## Competing interests

The authors declare that they have no competing interests.

## Authors’ contributions

OO, NU, BU and AA conceived the study and participated in the design and coordination, EE and NU wrote the first draft of the manuscript. All the authors revised and accepted the final manuscript.

## Pre-publication history

The pre-publication history for this paper can be accessed here:

http://www.biomedcentral.com/1471-2458/14/315/prepub
